# Comparison of *in vivo* pathogenicity of four *Candida auris* clades in a neutropenic bloodstream infection murine model

**DOI:** 10.1080/22221751.2020.1771218

**Published:** 2020-06-02

**Authors:** Lajos Forgács, Andrew M. Borman, Eszter Prépost, Zoltán Tóth, Gábor Kardos, Renátó Kovács, Adrien Szekely, Fruzsina Nagy, Ilona Kovacs, László Majoros

**Affiliations:** a Department of Medical Microbiology, Faculty of Medicine, University of Debrecen, Debrecen, Hungary; b Doctoral School of Pharmaceutical Sciences, University of Debrecen, Debrecen, Hungary; c UK National Mycology Reference Laboratory (MRL), Public Health England South-West, Bristol, UK; d Faculty of Pharmacy, University of Debrecen, Debrecen, Hungary; e Department of Pathology, Kenézy Gyula Hospital, University of Debrecen, Debrecen, Hungary

**Keywords:** Virulence, fungal tissue burden, aggregating *Candida auris*, myocardial involvement, contraction band necrosis

## Abstract

*Candida auris* is an emerging worldwide concern, but comparative data about the virulence of different *C. auris* lineages in mammalian hosts is lacking. Different isolates of the four prevalent *C. auris* clades (South Asian *n* = 5, East Asian *n* = 4, South African *n* = 5, and South American *n* = 5) were compared to assess their virulence in a neutropenic murine bloodstream infection model with *C. albicans* as reference. *C. auris*, regardless of clade, proved to be less virulent than *C. albicans.* Highest overall mortality at day 21 was observed for the South American clade (96%), followed by the South Asian (80%), South African (45%) and East Asian (44%) clades. Fungal burden results showed close correlation with lethality. Histopathological examination revealed large aggregates of blastoconidia and budding yeast cells in the hearts, kidneys and livers but not in the spleens. The myocardium of apparently healthy sacrificed mice as well as of mice found moribund showed contraction band necrosis in case of all lineages. Regardless of clade, the heart and kidneys were the most heavily affected organs. Isolates of the same clade showed differences in virulence in mice, but a markedly higher virulence of the South American clade was clearly demonstrated.

## Introduction

*Candida auris* was first identified in 2009 at a geriatric hospital in Japan and then in a nosocomial fungemia in Korea in 2011 [1,2]. Retrospective identification by DNA sequencing revealed the earliest known strain of *C. auris* was a bloodstream isolate from 1996 in a paediatric surgery patient in Korea, and the next earliest was found in 2008 in Pakistan [3]. In the last decade *C. auris* became an emerging organism with a public health significance causing asymptomatic colonization (skin, urine, indwelling devices) as well as sporadic hospital-acquired invasive infections (i.e. candidemia, intra-abdominal infections) worldwide [4]. Clinical studies showed that while *C. auris* affects both children and adults, it has been identified predominantly in critically ill patients in intensive care units [4–6]. Moreover, this pathogen is commonly multidrug resistant, posing a therapeutic challenge, however, susceptibility profiles may vary widely across isolates [4,7].

Five phylogenetically distinct clades (South Asian, East Asian, South African, South American and Iranian) of *C. auris* were identified based on whole genome sequence data of global clinical isolates [3,8]. Each clade differs from the other clades by >200,000 single-nucleotide polymorphisms, but isolates from the same clade are highly clonal, suggesting emergence of *C. auris* on all continents almost simultaneously.

The growing body of data suggests that significant differences exist between the five clades regarding their phenotypic characteristics and virulence [4,9,10]. In previous studies we have demonstrated that isolates belonging to South African and East Asian clades grew better in the presence of actidione, but do not produce even rudimentary pseudohyphae in Dalmau cultures as compared to South Asian clade [9]. The vast majority (84%) of South African isolates produce large aggregates in aqueous suspension on microscope slides and these isolates proved to be significantly less virulent in *Galleria mellonella* than the non-aggregating isolates of the South Asian lineage [10]. Moreover, isolates belonging to the South Asian, South African and South American clades but not the East Asian clade frequently cause outbreaks of invasive infections [4,11]. A single isolate representing a novel Iranian clade has been recently reported, but data on its pathogenicity and virulence is lacking [8].

Data about *in vivo* virulence of different *C. auris* clades in mammalian hosts is scant, the available studies focus mainly on the South Asian clade [12-17], moreover, the South African clade has never been examined. Fakhim et al. infected ICR outbred immunocompetent mice with inocula of 10^5^-, 10^6^ and 10^7^ CFU/mouse using two *C. auris* (both from the South Asian clade) and one *C. albicans* strains. They did not find statistically significant differences regarding lethality (60-80%) or fungal tissue burdens between *C. auris* and *C. albicans* [12]*.* In contrast, a single *C. auris* isolate from the South Asian clade proved to be significantly less virulent (0% lethality) than *C. albicans* (100% lethality) despite the 10-fold lower intravenous inocula of *C. albicans* in an immunocompetent BALB/c mouse model [13]. In another study using a temporarily neutropenic BALB/c murine model, 7 × 10^7^ CFU/mouse inoculated intravenously produced significantly shorter survival of mice with *C. albicans* than with the one *C. auris* isolate from the South Asian clade tested [14]. In a further study with two South Asian isolates even 10^8^ CFU/mouse did not cause death in immunocompetent BALB/c mice, but using immunosuppression with neutrophil-depleting antibodies (RB6-8C5 and 1A8) led to 100% lethality with the same dose [15]. Similar lethality (100%) was obtained by Singh et al. with a single South Asian isolate, using 5 × 10^7^ CFU/mouse inoculated intravenously in a deeply neutropenic CD-1 murine model [16].

Xin et al., intravenously infected four different mouse strains with single *C. auris* isolates from the East Asian and South American lineages. They did not find lethality in BALB/c, neutrophil elastase-deficient and C57BL/6 non-neutropenic mice and only a few deaths in neutropenic models even using an infectious dose as high as 2 × 10^8^ CFU/mouse. In contrast, the same dose produced 100% mortality within 2 days in A/J mice with C5 complement factor deficiency [17].

In this study we compared the behaviour of 17 clinical isolates derived from sterile and non-sterile body sites belonging to four *C. auris* lineages (South Asian, East Asian, South African, and South American) using a neutropenic murine bloodstream infection model. We also tested two additional *C. auris* isolates of environmental origin from the South American clade. As previous data from the *G. mellonella* model have suggested that at least some *C. auris* isolates showed virulence similar to *C. albicans*, we included two *C. albicans* bloodstream isolates in the same model for comparison [10].

## Materials and methods

### Isolates

Isolates of the four prevalent *C. auris* clades (South Asian *n* = 5, East Asian *n* = 4, South African *n* = 5, South American *n* = 5) tested, their origin and the origin of the *C. albicans* isolates are listed in Table 1. *C. auris* isolates were identified by a combination of ribosomal DNA gene sequencing targeting the 28S rRNA and/or ITS1 regions, which was also used for clade delineation [9,10]. *C. albicans* isolates were derived from a previous study [18]. Strains were stored at –70°C. Two days before the *in vivo* experiments, isolates were subcultured using Sabouraud agar and screened on CHROMagar Candida (Becton Dickinson) to ensure purity of *Candida* isolates.

**Table 1.** Characteristics of *Candida auris* and *Candida albicans* isolates used in the study. Bold indicates the isolates used in the fungal burden experiments.

Species and isolate number	Clade	Country	Body site	Aggregation
***C. auris* 196**	South Asian	Oman	Blood	**−**
*C. auris* 20 (NCPF 8985)	South Asian	England	Wound swab	**−**
***C. auris* 164**	South Asian	England	Swab	**−**
*C. auris* 10 (NCPF 8971)	South Asian	England	Wound swab	**−**
*C. auris* 27 (NCPF 89891)	South Asian	England	Pleural Fluid	**−**
*C. auris* type strain (NCPF 13029 = CBS 10913)	East Asian	Japan	External ear	**−**
***C. auris* 15** (NCPF 8984)	East Asian	Japan	External ear	**+**
*C. auris* 12372 (CBS 12372)	East Asian	Korea	blood	**+**
***C. auris* 12373 (CBS 12373)**	East Asian	Korea	blood	**+**
*C. auris* 185	South African	England	Blood	**+**
*C. auris* 228	South African	England	Skin swab	**+**
***C. auris* 206 (NCPF 13042)**	South African	England	Blood	**+**
***C. auris* 204**	South African	England	Tracheostomy	**+**
*C. auris* 2 (NCPF 8977)	South African	England	CSF	**+**
***C. auris* I-24**	South American	Israel	Blood	**−**
*C. auris* I-156	South American	Israel	Blood	**−**
*C. auris* I-172	South American	Israel	Blood	**−**
*C. auris* 13108				
(CDC B-13108)	South American	Panama	Hospital environment	**−**
***C. auris* 16565**				
**(CDC B-16565)**	South American	Chicago (from Colombia)	Hospital environment	**−**
***C. albicans* 3666**	Not applicable	Hungary	blood	Not applicable
*C. albicans* 2606	Not applicable	Hungary	blood	Not applicable

### Preparation of inocula

Yeast inocula were initially prepared in sterile saline, but in case of the South African isolates large, visible and highly stable aggregates were observed in the tubes or in the syringes at 10^6^ and 10^7^ CFU/mL; homogeneous suspensions were never obtained in saline. Aggregate formation was never observed with the other three *C. auris* clades nor with *C. albicans* isolates. Thus, to avoid aggregate formation, inocula were prepared in phosphate-buffered saline (PBS) and washed three times.

### Mice and immunosuppression

In our preliminary experiments with immunocompetent BALB/c mice using inoculum densities of 10^6^ and 10^7^ CFU *C. auris* per mouse prepared in PBS all mice survived. With the isolates of the South African clade aggregating in saline (but not with the three aggregating isolates from the East Asian lineage, Table 1), infectious doses >10^7^ CFU/mice produced early mortality (within one day) in 10-40% of mice, most probably due to embolization by fungal aggregates, which were either too small to be visible to the naked eye or produced *in vivo*. As early mortality was never observed at lower cell densities, we abandoned the immunocompetent model and used an immunocompromised murine model with the lower 10^7^ CFU/mouse inoculum for all four lineages to allow robust comparisons.

BALB/c male mice (23–25 g) were given cyclophosphamide 4 days before infection (150 mg/kg), 1 day before infection (100 mg/kg), 2 and 5 days post-infection (100 mg/kg) in the fungal tissue burden experiments; in the lethality experiments, immunosuppression was continued by administration of 100 mg/kg cyclophosphamide every third day until the end of the experiment at the 21st day [18]. The Guidelines for the Care and Use of Laboratory Animals were strictly followed during maintenance of the animals; experiments were approved by the Animal Care Committee of the University of Debrecen (permission no. 12/2014).

### Lethality experiments

Mice (groups of 10 mice/isolate) were infected intravenously through the lateral tail vein (day 0). The infectious dose for *C. auris* and *C. albicans* were 10^7^ and 10^5^ CFU/mouse, respectively, in volumes of 0.2 mL. Inoculum densities were confirmed by plating serial dilutions on Sabouraud agar plates.

Mice were monitored twice daily for lethality for 21 days. Animals that became immobile or showed signs of severe illness were terminated and recorded as dying on the following day. Dead animals (1–2 from each clade) were dissected for histopathology. Survival rates were compared within the same clade using the Kaplan–Meier log rank test; then data of isolates of the same clade were aggregated and survival was also analysed between different fungal clades [18]. Statistical tests were performed in Graph Pad 6.0.3.

### Fungal kidney tissue burden experiments

The infectious doses for *C. auris* and *C. albicans* were 10^7^ and 5 × 10^4^ CFU/mouse, respectively. Two *C. auris* isolates of each clade and one *C. albicans* isolate were chosen based on the lethality experiments (Table 1). One group included 11 mice. On day 2 and 6, 5 mice were sacrificed; both kidneys, the heart, the liver and the spleen were removed from each animal, weighed and homogenized aseptically in 1 ml of saline; the resulting tissue suspension was serially diluted. Fungal tissue burden was determined by quantitative culturing. The lower limit of detection was 100 CFU/g of tissue [18].

Mean fungal tissue burdens produced by different isolates/clades in the same organs on day 2 or 6 were compared using the Kruskal–Wallis test with Dunn’s post-test. *T*-test (with Welch’s correction, where appropriate) was used to compare the mean fungal tissue burdens at day 2 with day 6 for the same organ with each isolate [18].

### Histopathology

Two mice from two different groups representing each clade (eight isolates altogether) derived from the fungal tissue burden experiments were used for histopathological examination on day 6. Seven mice freshly found dead in the lethality experiment (infected with *C. auris* type strain (NCPF 13029=CBS 10913), isolates CBS 12372, 2 (NCPF 8977), 27 (NCPF 89891), I-24, I-172, and CDC B-13108) were dissected and analysed similarly. Moreover, on day 1 a single mouse infected with isolates 196, CBS 12373, 204 and I-24 (representing each clade) were used to determine the early heart involvement. Organs (heart, both kidneys, liver and spleen) were fixed in formalin and embedded in paraffin. Tissue sections (4 µm) were stained with haematoxylin-eosin and Periodic Acid Schiff (PAS). Hearts were stained with Mallory’s phosphotungstic acid haematoxylin (PTAH) as well.

## Results

### Lethality

#### Candida auris

**Figure 1.**
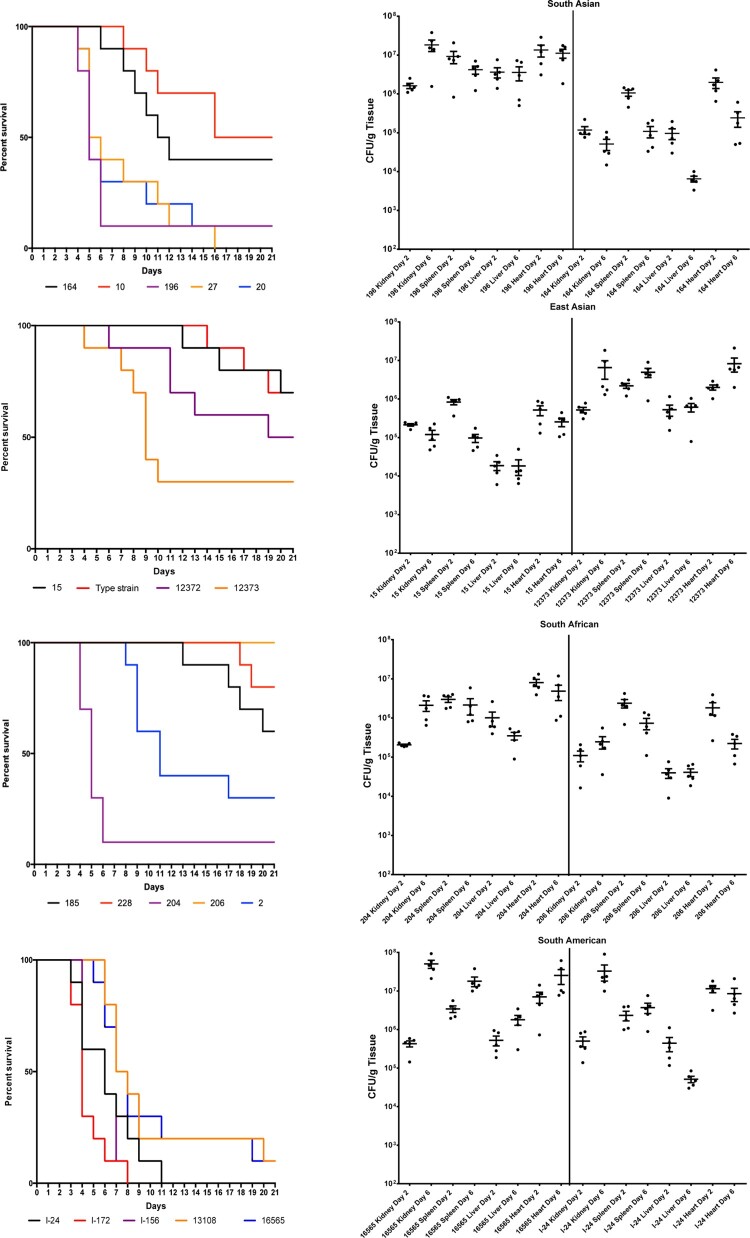
Survival (left) and fungal tissue burden (right) of neutropenic BALB/c mice infected with the four *Candida auris* clades. In survival studies mice were infected with 19 isolates (South Asian *n* = 5, East Asian *n* = 4, South African *n* = 5 and South American *n* = 5) representing the four clades. In the graph representing the East Asian clade the “Type strain” represents NCPF 13029 (CBS 10913) type strain isolated from external ear. Fungal kidney, spleen, liver and heart burdens were determined with isolates 196 and 164 from the South Asian, isolates 15 (NCPF 8984) and 12373 (CBS 12373) from the East Asian, isolates 206 (NCPF 13042) and 204 from the South African and isolates I-24 and 16565 (CDC B-16565) from the South American clades at days 2 and 6. The bars represent the medians.

Mice infected with different *C. auris* isolates, with the exception of the isolates from Israel (South American clade), did not show clinical signs of systemic infection. Death occurred unexpectedly; one day before their death mice appeared clinically normal. Torticollis or other signs of the central nervous system involvement were never observed. Highest overall mortality at day 21 was observed for the South American clade (96%, range 90%-100%), followed by South Asian isolates (80%, range 50–100%), while infection with South African and East Asian isolates resulted in markedly lower cumulative lethality (45% and 44%, ranges 0–90% and 30–70%, respectively) (Figures 1 and S1). Survival differed significantly within clades (*P* = 0.0005, *P* = 0.0010, *P* < 0.0001 and *P* = 0.0255 for South American, South Asian, South African and East Asian clades, respectively). In cases of the South American and South Asian clades mortality was first observed at days 3 and 4, respectively. All mice inoculated with isolates from Israel (South American clade) died by day 11. In contrast, late onset of mortality (later than day 8) was typical for South African and East Asian clades (Figure 1). Notably, the two bloodstream isolates from the East Asian clade caused higher mortality (50–70% vs. 30%) than isolates from external ear samples. However, isolate 206 (a bloodstream isolate from the South African clade) did not kill any mice during the 21-day experiment.

#### Candida albicans

**Figure 2.**
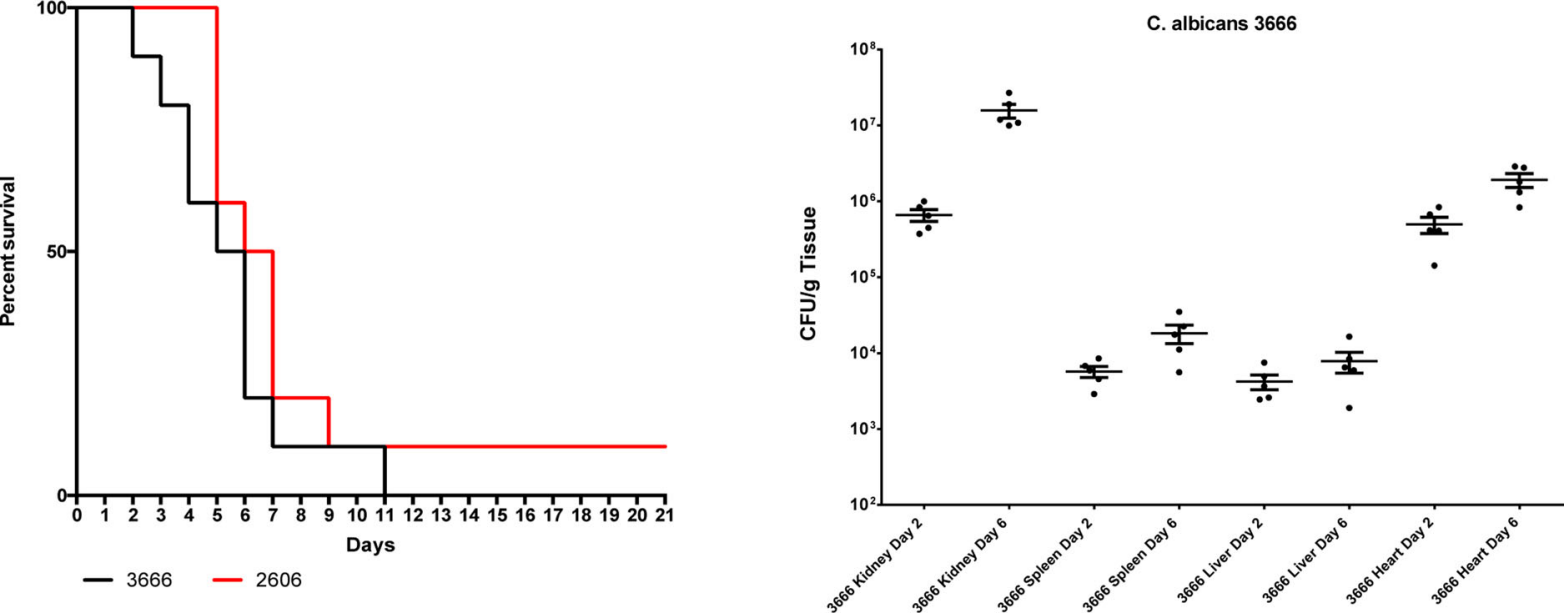
Survival (left) of neutropenic BALB/c mice infected with *Candida albicans* isolates 3666 and 2606. Fungal kidney, spleen, liver and heart burdens (right) was determined with isolate 3666 at days 2 and 6. The bars represent the medians.

The two *C. albicans* isolates using a 100-fold lower inoculum than *C. auris* produced 90–100% mortality rates with typical signs (lethargy, loss of appetite, hunched back, and ruffled fur) of systemic infection (*P* > 0.05) (Figure 2).

### Fungal organ tissue burden

#### Candida auris

Regardless of clade, the heart and kidneys were the most heavily affected organs. Fungal burden results showed close correlation with lethality, i.e. infection with isolates causing high mortality in the lethality experiments within each clade produced higher fungal tissue burdens (Figure 1). Six-day heart and kidney burdens with the isolates causing high lethality (≥90%) were at least one order of magnitude higher than burdens caused by isolates associated with intermediate or low lethality (30–60%). This was apparent even at day 2 in case of heart burdens. Kidney and heart burdens tended to increase from day 2 to day 6 in case of highly lethal isolates, but not for isolates causing intermediate-low lethality. In case of the South Asian, East Asian and South African clades, the lower-lethality isolates produced lower fungal burdens, highly lethal isolates produced higher burdens, while in case of the South American clade both tested isolates produced high lethality and high burdens (Figure 1).

Spleen and liver burdens were more variable. Mean fungal spleen burdens at day 2 did not differ significantly between isolates, but at day 6 CFU number of the isolate 16,565 (South American clade) was significantly higher than those for isolates 15 (NCPF 8984) and 164 (East Asian and South Asian clades, respectively) (*P* < 0.001) (Figure 1). Mean fungal liver burdens were the lowest on both tested days compared to other examined organs; the highest (3.6 × 10^6^ CFU/g) and lowest (6.5 × 10^3^ CFU/g) mean fungal burdens were noticed in cases of high-lethality isolates 196 and intermediate-lethality 164, respectively, (both belonging to South Asian lineage) at day 2.

#### Candida albicans

Fungal tissues burden increases with isolate 3666 were statistically significant in cases of heart, kidneys and spleen (*P* = 0.016, *P* = 0.008 and *P* = 0.008, respectively) from day 2 to day 6. Mean fungal liver burdens at days 2 and 6 were lower than 10^4^ CFU/g (Figure 2).

Mean fungal heart and kidney burdens were comparable to those with high-lethality *C. auris* isolates at day 6, but were higher than in case of *C. auris* isolates causing intermediate or low lethality. However, mean liver fungal burden was significantly lower than isolates 16,565 and 196 of *C. auris* (both being high-lethality isolates) (Figures 1 and 2).

### Histopathology

#### Candida auris

**Figure 3.**
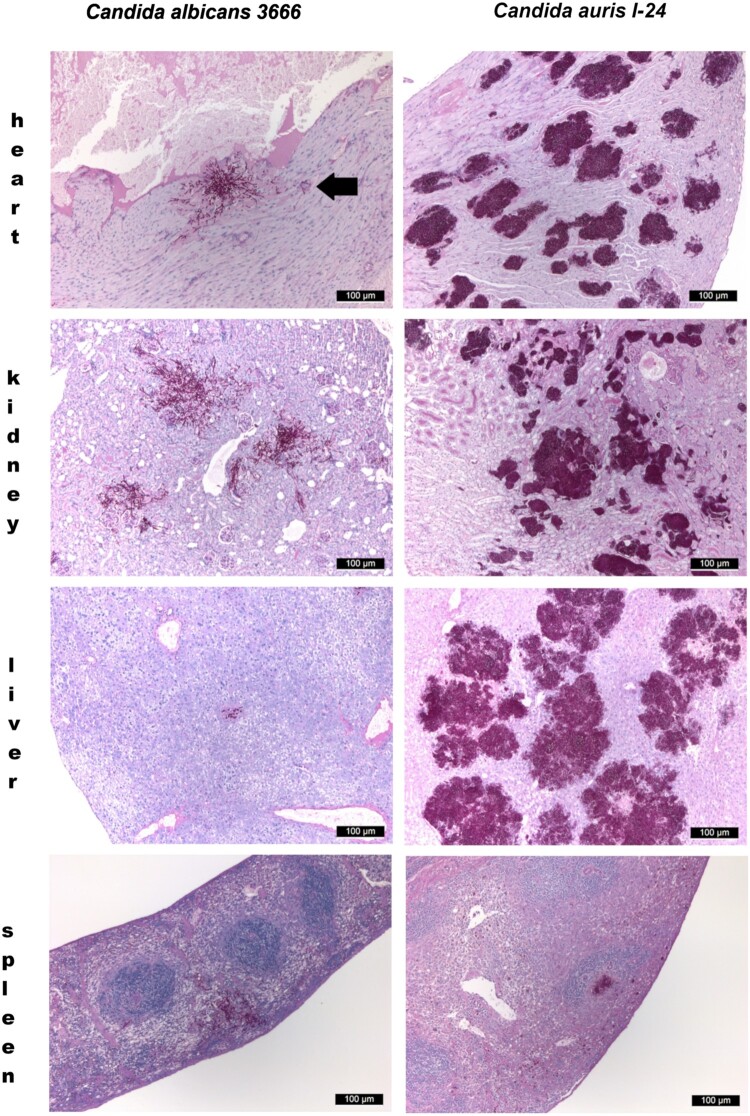
Histopathological examination of the heart, kidney, liver and spleen using Periodic Acid Schiff staining from neutropenic mice intravenously challenged with *Candida albicans* isolate 3666 (left panels) and *C. auris* isolate I-24 (right panels), respectively. Histopathological examination was performed six days post infection. In case of *C. albicans* in the heart, in the kidney and in the spleen blastoconidia and budding yeast cells, pseudohyphae and hyphae were seen. In the liver hyphae were not detected. In the heart both endo- and myocardial involvement is noticeable, the subendocardial myocardium is affected most markedly. Signs of beginning blood vessel invasion in the myocardium is detectable (black arrow). In the kidney *C. albicans* was detected within the parenchyma, tubuli and glomeruli. In the spleen pseudohyphae and hyphae were seen in the red pulp. *C. auris* produced large aggregates in the heart, kidney and liver with numerous blastoconidia and budding yeast cells were detected. In the heart coagulative necrosis of myocytes was noticed. In the kidney *C. auris* cells were detected in the parenchyma, tubuli but not in the glomeruli. In the liver dilated liver sinusoids were filled with yeast cells with central necrosis of lobuli and vacuolar degeneration of hepatocytes. In the spleen yeast cells were seen at the border of the red and white pulp with focal destruction of the white pulp. Small fungal lesions were detectable under the capsule of the spleen and in the sinuses. Magnification × 100.

Only single yeast cells and numerous budding yeast cells were produced in the organs, pseudohyphae were never observed. However, all isolates produced numerous, large aggregates in all examined organs with the exception of the spleen, both in mice dissected during the fungal burden experiments and in mice found dead in the lethality experiments (Figure 3).

**Figure 4.**
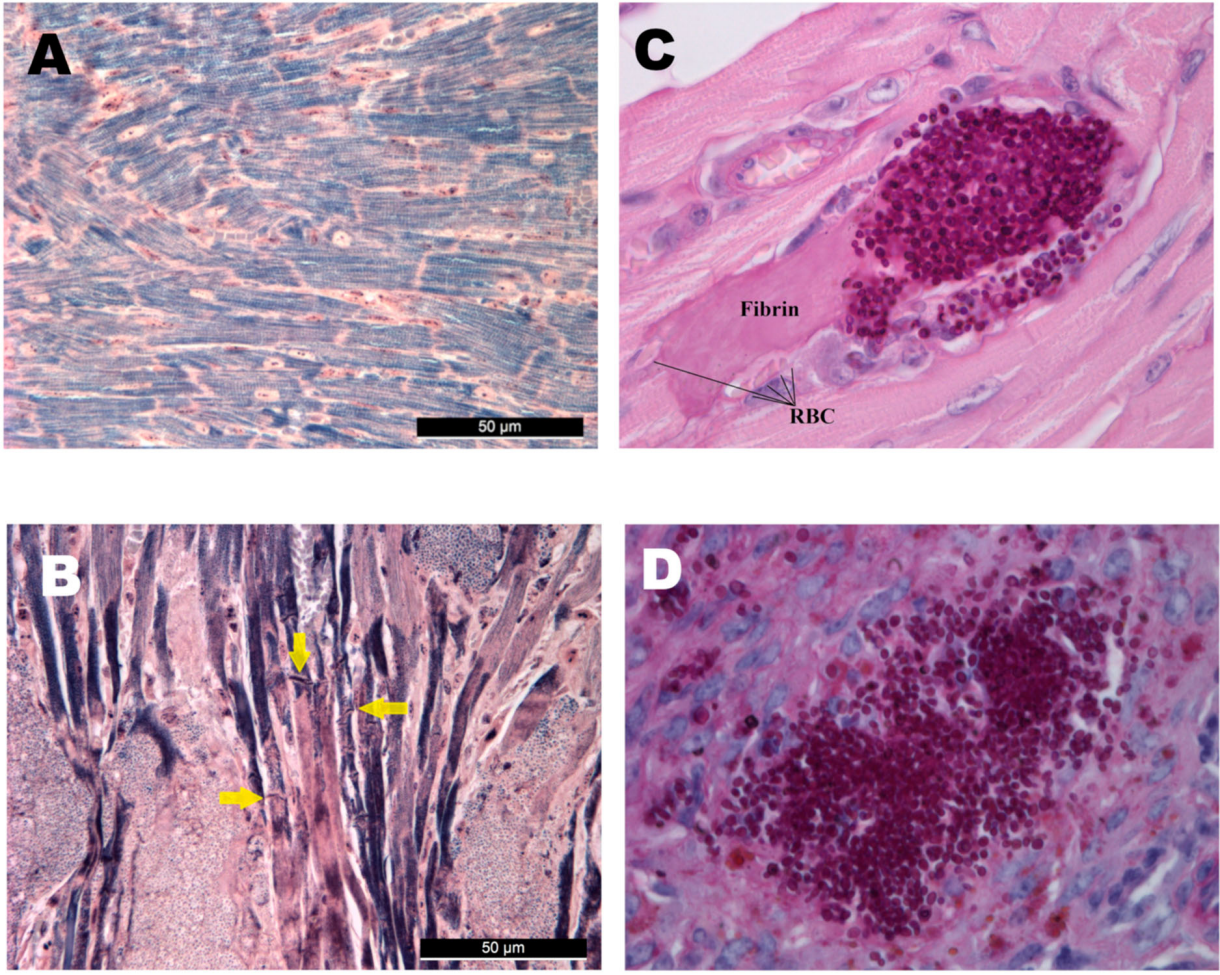
Histological findings from neutropenic mice intravenously challenged with *Candida auris.* Normal (A) and damaged heart (B) with Mallory’s PTAH staining. PTAH staining performed on the heart of a moribund dissected mouse on day 5 infected with isolate 196 (B) revealed contraction band necrosis or myofibrillar degeneration (yellow arrows). Morphology of *C. auris* with Periodic Acid Schiff (PAS) staining in the heart’s arterioles on day 1 post infection (C), and in the spleen (D). PAS staining showed blastoconidia and budding yeast cells but never pseudohyphae and hyphae (C–D). Magnification, A-B × 400, C × 1000, D × 400, RBC; red blood cells.

All examined hearts (regardless of clade) exhibited multiple foci of abundant yeast cells between myofibres with coagulative necrosis of myocytes (Figure 3). Myofibres were frequently distorted by the fungal lesions. Mallory’s PTAH staining revealed that myocardial fibres lost their cross-striations, confirming the contraction band necrosis or myofibrillar degeneration in all mice freshly found dead in the lethality experiments (Figure 4(A, B)) [19]. Contraction band necrosis was found in infected but apparently healthy mice dissected on day 6 as well. Early heart involvement on day 1 was confirmed by detecting clumps of blastoconidia and budding yeast cells filling the arterioles of the myocardium (Figure 4(C)). Foci appeared in the sub-endocardial myocardium and the pericardium only in the late stages of the infection.

In the kidneys, there was evidence of multifocal infiltration with *C. auris* within the parenchyma, with focal destruction of tubuli, but glomeruli were not affected. Necrotizing areas were frequently seen (Figure 3). In the livers *C. auris* produced large, multifocal lesions; the dilated liver sinusoids were filled with abundant yeast cells, fungi spread radially in the liver parenchyma with central necrosis of lobuli. Vacuolar degeneration of hepatocytes was commonly seen (Figure 3). In the spleens, in contrast, only small clumps of fungal cells were seen (Figures 3 and 4(D)).

#### Candida albicans

In the heart, kidneys and spleen abundant single and budding yeast cells, pseudohyphae and hyphae were seen (Figure 3). In the liver only a small number of blastoconidia and budding yeast cells and pseudohyphae were noticed (Figure 3). In the heart large necrotic areas were seen involving the endo- and myocardium but without contraction band necrosis (Figure 3).

## Discussion

The most common clinical presentation of *C. auris* infection is candidemia in intensive care units among critically ill patients. Major risk factors are diabetes mellitus, surgical cardiovascular and gastrointestinal diseases, haematological malignancies and corticosteroid therapy [3–6,20–23]. Interestingly, neutropenia is not considered as an important risk factor for invasive *C. auris* infection. This clinical experience may be related with the *in vitro* finding that *C. auris* triggers neutrophil engulfment and production of neutrophil extracellular traps less effectively compared to *C. albicans* [24]. However, Chowdhary et al. have reported 6 of 12 patients with *C. auris* candidemia with neutropenia [25]. Candidemia is frequently persistent in spite of apparently adequate targeted antifungal therapy; septic metastatic complications (i.e. spondylodiscitis, endo- and pericarditis) are also often encountered [2,4,22,23]. Kidneys are often involved in the pathogenesis as well, both in cases with or without candidemia [4–7,20-23,25,26]. The crude in-hospital mortality rate for *C. auris* candidemia may be as high as 68–80% (3,22). Attributable mortality due to *C. auris* is hard to determine due to the severe underlying conditions of the patients; limited available estimates fall between 22.2 and 66.6% [2,6,23,26,27].

Data on clade-specific mortality are scant. In the first reported cases of *C. auris* candidemia two of three patients infected with the East Asian clade died in Korea and showed persistent candidemia in spite of fluconazole and amphotericin B treatment [2]. Lockhart *et al*. reported 47–72%, 60% and 33% mortality rate with South Asian, South American and South African clades, respectively [3].

Our lethality results in a deeply neutropenic murine model were consistent with other groups showing that *C. auris*, regardless of clade, is less virulent than *C. albicans* [13,14,17]. In this study, the ranking in the virulence of the four *C. auris* clades was South American > South Asian > South African = East Asian (Figures 1, 2 and S1). Similar high virulence was reported by Xin et al. in A/J (100% lethality) but not in BALB/c neutropenic mice (40% lethality) using a single South American isolate [17]. Our lethality results with the South Asian clade (50–100% lethality) correlate well to those previously reported for neutropenic BALB/c or CD-1 mice (40–100% lethality) with the same or higher (from 10^7^ to 10^8^ CFU/mouse) infectious doses [15,16]. The lack of similar studies with the South African clade precludes comparison to our results. However, we confirmed the previous observation in a *G. mellonella* model that aggregating South-African *C. auris* isolates are less virulent than non-aggregating South-African isolates [10]. Moreover, we found significant differences in virulence among isolates of the same clade in case of virtually all clades. These results are concordant with the previous *in vitro* findings that *C. auris* isolates may produce several virulence factors in a strain-dependent manner but to a lower extent than *C. albicans* [4,28]. Notably, the *C. auris* isolates from the South American clade originating from bloodstream infection and the hospital environment produced very similar mortality rates at day 21 (100% and 90%, respectively). This highlights that virulent *C. auris* persists for long periods in the hospital environment and these isolates are potential sources of colonization and infection for high-risk patients [3–5,23,27].

High fungal tissue burdens supported our histopathological findings regardless of clade; at day six multifocal, large aggregates of single and budding yeast cells were always found in the hearts, kidneys and livers but not in the spleens (Figure 3). Interestingly, these lesions were detected both with aggregating and non-aggregating isolates, suggesting that both phenotypes behave similarly *in vivo*. These large yeast cell aggregates in tissues may shield the fungus from the effective immune response and promote fungal persistence and replication [4,10,11]. Moreover, it cannot be ruled out that homogenization of these large tissue aggregates was not perfectly successful and despite homogenization, some cells might have remained aggregated, thus the CFU numbers may have been underestimated. This may explain why some isolates (i.e. isolate 164 from the South Asian clade) showed unexpectedly less CFU on day 6 than day 2.

Large, multifocal lesions in the myocardium without the involvement of the endocardium suggest hematogenous seeding; on day 1 abundant single and budding yeast cells were seen in the coronary arteriolae in mice infected with all *C. auris* lineages (Figure 4(C)). Our results are in accordance with others who observed high fungal burdens (≥×10^5^ CFU/g) in the myocardium with BALB/c, A/J, neutrophil elastase-deficient and C57BL/6 neutropenic and non-neutropenic mice infected with *C. auris* isolates from the South American and South Asian clades [15,17]. In contrast, with the more virulent *C. albicans* endocardial involvement was found and myocardial involvement was primarily subendocardial, suggesting a penetration of the heart muscle by yeasts starting from the endocardium.

In spite of the high fungal burden in the heart even at day 2 (≥5.2 × 10^5^ CFU/g), increased early mortality was not observed with the less virulent isolates from the South African, East Asian and South Asian lineages. However, the heavy myocardial fungal burden is associated with irreversible damage of the myocardial cells as detected by the contraction band necrosis (Figure 4(B)) found in apparently healthy dissected mice as well as in mice freshly found dead. Translating our results to clinical situations, delay in the diagnosis and treatment of disseminated *C. auris* candidiasis may increase the risk for fungus-induced myocardial failure and sudden death.

High fungal kidney and/or heart burdens are frequently associated with heavy fungal burden in the brain and lungs as reported both in immunocompetent and neutropenic mice. Singh et al., noticed simultaneously heavy fungal kidney (2 × 10^8^ CFU/g) and brain (2 × 10^6^ CFU/g) burdens. Interestingly, *C. auris* cells found in the brain were localized mainly in the capillaries rather than in the cerebral tissue itself [16]. *Candida* pneumonia is a rare manifestation of invasive *Candida* infections in humans, but in immunocompetent ICR and BALB/c mice higher infectious doses (≥10^7^ CFU/g) produced 10^3^–>10^5^ CFU/g lungs tissue burdens [12,13]. Although in this study we did not examine the fungal burdens in the brain and lungs, based on the high fungal burdens in the hearts and kidneys, central nervous system and lung invasion is also feasible. However, we did not observe signs of meningeal involvement [15].

To our best knowledge this is the first study to compare the *in vivo* virulence of the four *C. auris* lineages distributed worldwide in a mammalian host. We observed high fungal burdens in organs, especially in the myocardium, confirming that the heart is a major target in cases of disseminated *C. auris* infection. However, failure of other organs especially the kidney may contribute to mortality as well. Isolates of the same clade showed differences in murine virulence; a markedly higher virulence of the South American clade was clearly demonstrated both in case of bloodstream and hospital environmental isolates.

## Supplementary Material

Supplemental Material
