# Supplementary figures and images for: Comparison of in vivo pathogenicity of four Candida auris clades in a neutropenic bloodstream infection murine model
Source: Emerg Microbes Infect. 2020 Jun 2;9(1):1160–9. doi: 10.1080/22221751.2020.1771218 (PMC7448943; doi:10.1080/22221751.2020.1771218)

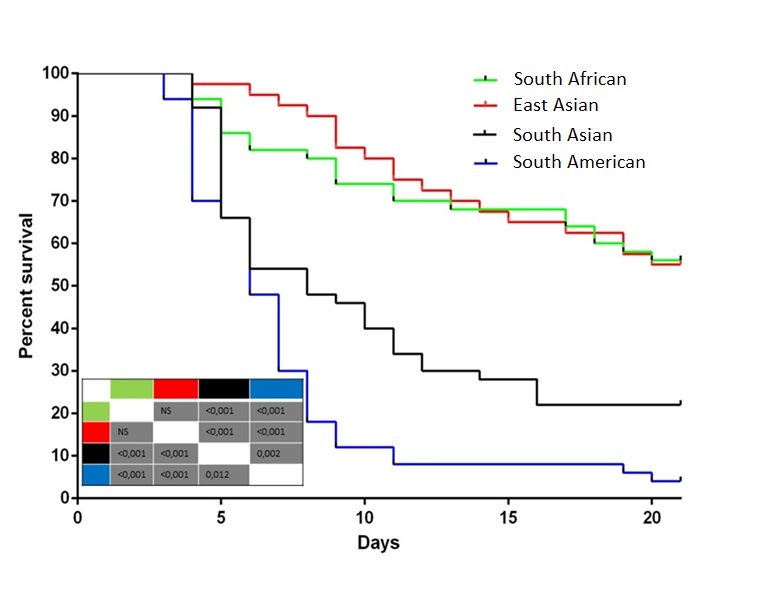

Supplement: Supplemental Material [file TEMI_A_1771218_SM2291.docx]
